# A case report of a bleeding case after removal of chest drain after lung surgery

**DOI:** 10.1097/MD.0000000000039279

**Published:** 2024-08-30

**Authors:** Qichen Liang, Baoyu He, Bin Zhang, Ziteng Zhang

**Affiliations:** a School of Clinical Medicine, Jining Medical College, Jining, Shandong, P. R. China; b Department of Laboratory Medicine, Affiliated Hospital of Jining Medical University, Jining Medical University, Jining, Shandong, P. R. China; c Department of Thoracic Surgery, Affiliated Hospital of Jining Medical University, Jining, Shandong, P. R. China; d Department of Thoracic Surgery, Qinghai Red Cross Hospital, Xining, Qinghai, P. R. China.

**Keywords:** case report, hemorrhagic shock, intrathoracic drain, postoperative hemorrhage, thoracoscopic lobectomy

## Abstract

**Rationale:**

Postoperative bleeding after lobectomy is relatively rare. By analyzing and discussing the case history and management of hemorrhagic shock caused by chest tube removal after lobectomy, we can achieve the purpose of preventing postoperative bleeding after thoracic surgery and reducing postoperative complications, which can help avoid the risk of second surgery, shorten the patient’s hospital stay, reduce the cost of medical care, and improve the patient’s quality of life.

**Patient concerns:**

A case of bleeding from tube removal after lobectomy. The bleeding from chest drain removal on the 3rd day after thoracoscopic lobectomy resulted in hemorrhagic shock, which was stopped by thoracoscopic exploration again under active antishock, and there was no recurrence of bleeding after the operation, and the patient was discharged from the hospital after chest drain removal.

**Diagnoses:**

Enhanced computed tomography of the chest revealed a space-occupying lesion in the middle lobe of the right lung.

**Interventions:**

Thoracoscopy was performed again on the condition of active anti-shock.

**Outcomes:**

On the third day after thoracoscopic lobectomy, the patient underwent removal of the chest drain and subsequently experienced hemorrhagic shock. Given the necessity of maintaining anti-shock measures, the patient was subjected to a second thoracoscopic exploration with the objective of halting the hemorrhage. Following this procedure, the patient did not present with any further episodes of bleeding. Subsequently, a new chest drain was placed, and once the drainage flow had diminished to an acceptable level, the chest drain was removed. The patient subsequently made a full recovery and was discharged from the hospital.

**Lessons:**

Even if the safely inserted drain tube is removed, the thoracic surgeon must be aware of possible vascular bleeding.

## 1. Introduction

Lobectomy is currently one of the most common surgical treatments for malignant and nonmalignant lung diseases, and postoperative hemorrhage is a major but rare complication, which can be life-threatening if not diagnosed in time and managed correctly. An indwelling chest drain is often required after lung surgery to drain intrathoracic air and fluid and to accelerate recovery. Here, we report a case of bleeding from tube removal after lobectomy. The bleeding from chest drain removal on the 3rd day after thoracoscopic lobectomy resulted in hemorrhagic shock, which was stopped by thoracoscopic exploration again under active antishock, and there was no recurrence of bleeding after the operation, and the patient was discharged from the hospital after chest drain removal. The purpose of this case is to analyze and discuss the case of hemorrhagic shock caused by removal of chest drain after lobectomy. To prevent bleeding after thoracic surgery, reduce postoperative complications, and optimize the diagnostic and therapeutic process.

## 2. Case report

### 2.1. History

A 25-year-old female patient was admitted to the Department of Thoracic Surgery of the Affiliated Hospital of Jining Medical College on November 29, 2023 with the main cause of “blood in sputum for more than 5 months.” The patient had blood in sputum 5 months ago, which occurred every month, with a small amount of dark-red clots, no chest tightness or wheezing, no fever, and no respiratory distress. Enhanced computed tomography (CT) of the chest revealed a space-occupying lesion in the middle lobe of the right lung, and he was admitted to the Department of Thoracic Surgery of the Affiliated Hospital of Jining Medical College for surgical treatment.

### 2.2. Physical examination

T: 36.3 °C, P: 91 beats/min, R: 20 beats/min, BP: 110/67 mm Hg, the patient’s consciousness was clear, normal spirit, bilateral supraclavicular lymph nodes were not touched, the trachea was centered, the thorax was symmetrical, bilateral respiratory movement was symmetrical, there was no pleural friction, no subcutaneous emphysema, lung percussion was clear, the respiratory sound of the right lung was normal, there was no rales, HR: 91 beats/min, the heart rhythm was regular. No pathological murmur was heard.

### 2.3. Ancillary examination

**Figure 1.**
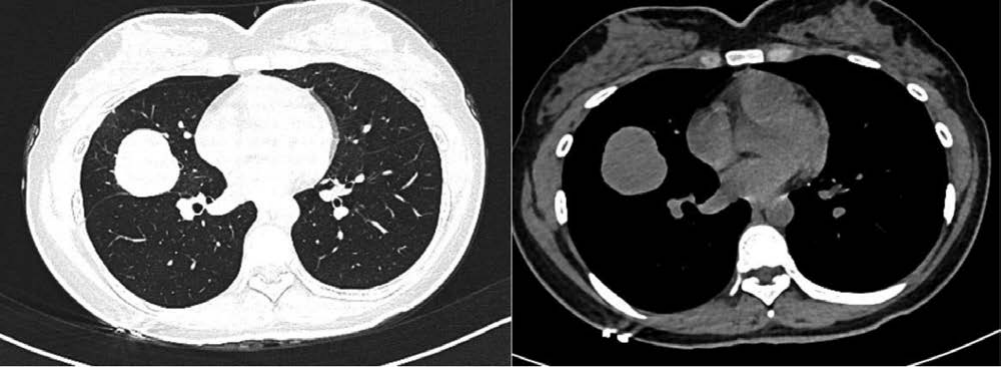
November 29, 2023 chest CT. CT = computed tomography.

November 29, 2023 chest CT (Fig. 1): (1) occupational lesion in the middle lobe of the right lung with bronchial truncation in the lateral segment of the middle lobe; (2) little chronic inflammation in the middle lobe of the right lung; and (3) otter-tailed liver with small intrahepatic cysts.

## 3. Diagnosis and treatment

**Figure 2.**
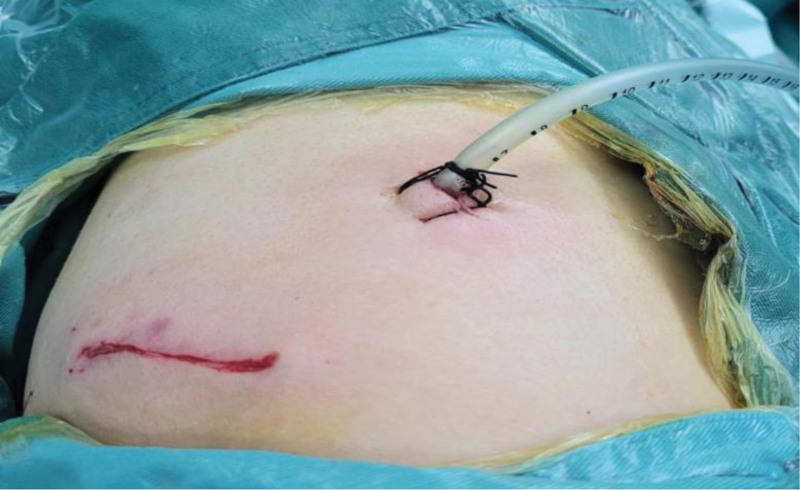
The drain tube was placed after the surgery.

**Figure 3.**
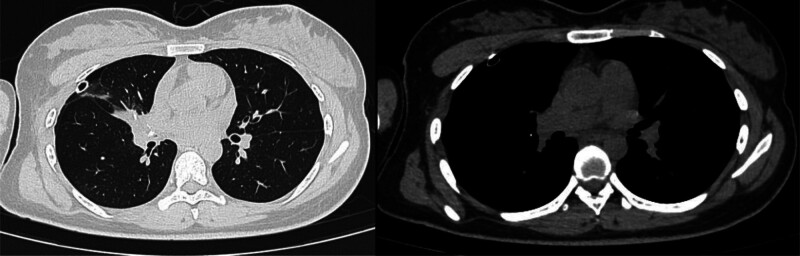
December 05, 2023 chest CT. CT = computed tomography.

According to the patient’s medical history, physical examination, and auxiliary examination results, the diagnosis was pulmonary occupational lesion (right middle). Thoracoscopic lobectomy (right middle) was performed under general anesthesia with complex nerve block anesthesia on December 02, 2023 at 11:30. Surgical record: bronchial examination was performed before tracheal intubation during the operation, and no abnormality was seen, 3 cm was taken in the 4th intercostal space of the right anterior axillary line as the operation hole, and the 7th intercostal space of the mid-axillary line was about 1 cm in length as the observation hole, and the adhesion band was seen in the thoracic cavity, and the tumor was found to be located in the middle lobe of the right lung, with the size of about 5 × 4 × 4 cm, and the texture was soft and complete resection of the right middle lobe of the right lung, and after the operation the lung was puffed up, and there was no leakage of air in the stumps of the bronchus. One drain was placed (shown in Fig. 2), and the operation went smoothly, with an operating time of 85 minutes, intraoperative bleeding of about 10 mL, no blood transfusion, and the patient returned to the ward after the operation. December 03, 2023 7:48 check: postoperative day 1, pain at the incision, score 2, no chest tightness, shortness of breath, no coughing up blood, cardiac monitoring prompts: P: 76 beats/min, BP: 130/81 mm Hg, oxygen saturation: 98%. Closed chest drain was smooth, no obvious gas drainage, and 50 mL of light red fluid was drained. December 04, 2023 patient’s 2nd postoperative day, pain at the incision, score 3, closed chest drain was smooth, and about 150 mL of light yellow fluid was drained. December 05, 2023 10:41 checkup: on the 3rd postoperative day, review of the chest CT (Fig. 3) showed: (1) after partial resection of the right middle lobe of the lungs, (2) a little inflammation in the lower lobes of both lungs, and a small amount of fluid in the left pleural cavity (3) otter-tailed liver with small intrahepatic cysts.

**Figure 4.**
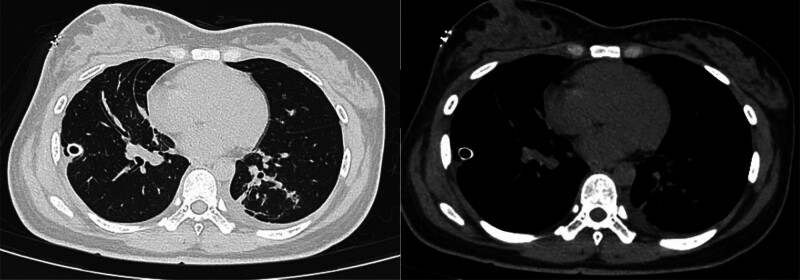
December 11, 2023 chest CT. CT = computed tomography.

Thoracic drainage flow gradually reduced, review chest CT did not see obvious pneumoperitoneum and effusion, lung reopening is good, thoracic drainage tube removed today, at 9:00 am this morning to remove the thoracic drainage tube. 12:45: the patient was covered with cold sweat, immediately check the patient, chest incision healing well, no redness, swelling and oozing, thoracic closed drainage tube has been removed. Oxygen and cardiac monitoring were given, and an urgent electrocardiograph was checked for (1) sinus tachycardia: (2) mild ST segment changes. Glucose sodium chloride injection 500 mL rapid drip was given. The above symptoms did not significantly improve, transferred to the monitoring room. 13:46 received point of care testing critical value report: blood glucose 31.80, hemoglobin 54 g/L, analysis of the cause of hemorrhagic shock, immediately performed bedside chest ultrasound, suggesting that the right side of the thoracic cavity mixed density shadows, blood clots can be seen, given 2 U of suspension of red blood cells and 500 mL of plasma into the. 14:33: the patient’s face is pale, the whole body is wet and cold, irritability obvious, nodding-like breathing. Blood pressure was about 90/60 mm Hg, heart rate was 130 beats/min, blood gas analysis showed that blood pH was 7.44, oxygen partial pressure was 157 mm Hg, hemoglobin was 54 g/L, and it was diagnosed as “hemorrhagic shock,” and the patient was given an open venous access, a large amount of rehydration, and norepinephrine was pumped in to maintain blood pressure, and the patient’s conscious state turned into a coma. After the patient’s conscious state turned into coma, apnea appeared, and he was given transoral intubation with ventilator to assist respiration, and at the same time, he was given virus-inactivated frozen plasma of 500 mL, 10 units of leucocyte-suspended erythrocytes, and 10 units of cold-precipitated clotting factor. Correction of shock was accompanied by thoracoscopic exploration at 16:10 under resting compound anesthesia (right),the exploration saw about 2000 mL of blood accumulation in the chest cavity, saw active bleeding at the pleural adhesion of the chest wall in the first operation, free along the chest wall outside to the intercostal space opposite to the bleeding point, immediately gave ligation to stop the bleeding, cleaned up the blood accumulation in the chest cavity, repeated saline rinsing of the chest cavity, puffed up the lungs after the operation, and placed one drain, the operation went smoothly, and the operation lasted 90 minutes. December 06, 2023 the patient’s vital signs were relatively stable, sinus rhythm, heart rate 47 to 129 beats/min, respiration 14 to 25 breaths/min, blood pressure 79 to 162/52 to 123 mm Hg, body temperature 36.2 to 36.6 °C, general state was OK, clear, poor mental, both lungs respiratory sounds were thick, and a small amount of wet rhonchi was audible. December 07, 2023 the patient developed pain. The pain was confined to the lateral chest wall with a score of 2, the closed chest drainage tube was patent, draining about 320 mL of light red fluid. December 08, 2023 the patient’s closed chest drainage tube was patent, draining about 280 mL of light red fluid. December 09, 2023 the patient’s closed chest drainage tube was patent, draining about 200 mL of light yellow fluid. December 10, 2023 the patient’s closed chest drainage tube was patent, draining about 200 mL of light yellow fluid. December 10, 2023 the patient’s closed chest drainage tube was patent, draining about 200 mL of light yellow fluid. December 11, 2023 the patient recovered well after surgery, the chest tube drainage flow gradually decreased, the chest CT was not obvious pneumoperitoneum and fluid accumulation (as shown in Fig. 4), the lung reopening was good, the chest tube was removed today, and the patient continued to be observed for changes in her condition. December 13, 2023 the patient did not have fever, chest tightness, shortness of breath, mild cough, small amount of sputum, and her chest incisions were healing well, with no oozing redness or swelling, the chest incision is healing well, no oozing redness and swelling, the chest tube has been pulled out without complaining of discomfort, and there is no change in condition.

The patient provided written informed consent for the publication of this case report.

## 4. Discussion

Surgical resection is currently the mainstay of surgical treatment for malignant or nonmalignant lesions in the chest.^[1]^ And video-assisted thoracoscopic lobectomy (VL) is usually used as the main surgical procedure in clinical practice. VL has a lower complication rate, shorter hospital stay, and faster postoperative recovery compared to traditional open lobectomy (OL).^[2]^ A chest drain is often required to be placed in the postoperative period of VL, which is used to drain postoperative gas and blood produced in the chest to restore normal intrathoracic pressure, help lung reopening, and prevent intrathoracic infection.^[3,4]^ Common postoperative pulmonary complications of VL often include: intrapulmonary pneumonia, pulmonary atelectasis, and persistent air leakage,^[5,6]^ whereas thoracic hemorrhage is an infrequent but highly dangerous complication of VL, and thoracic hemorrhage is even rarer after extubation, it is difficult to detect when the bleeding is small, and the patient is already in a state of shock when the bleeding is large, in addition, it is difficult to stop the bleeding by the usual normal hemostasis, and the large amount of bleeding and its duration are very likely to cause serious bleeding or hemothorax and shock.^[7]^ Hemothorax is an uncommon but extremely dangerous clinical condition caused by rapid respiratory failure and massive insidious blood loss into the pleural cavity, which is one of the reasons for unexplained significant hypovolemia after thoracic surgery.^[8]^ Common causes of thoracic hemorrhage are bronchial arterial hemorrhage, hemorrhage from large vessel stumps and bronchial stumps, lung parenchyma hemorrhage, chest wall incision hemorrhage, and intrathoracic hemorrhage,^[9]^ of which intrathoracic hemorrhage is commonly seen in patients with pleural adhesions.

**Figure 5.**
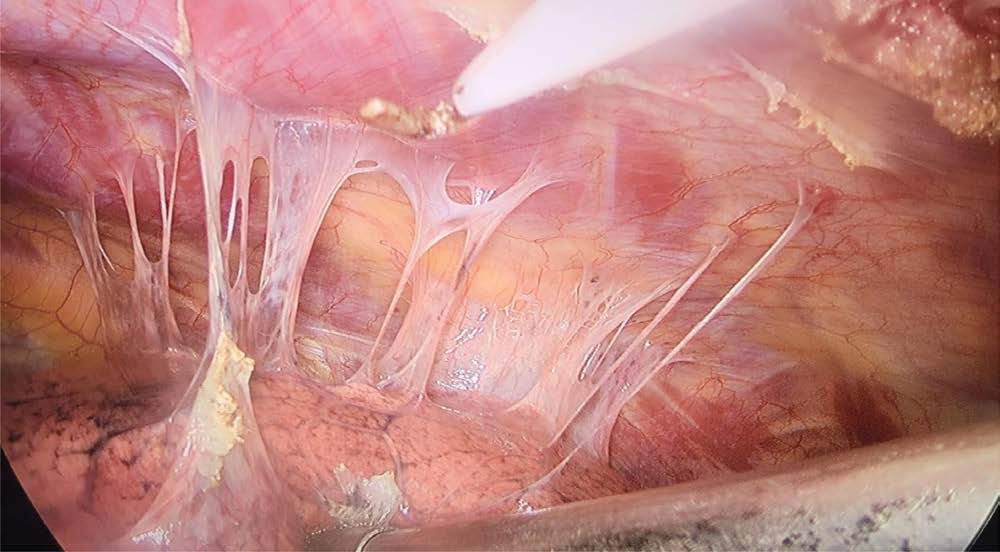
Pleural adhesion.

**Figure 6.**
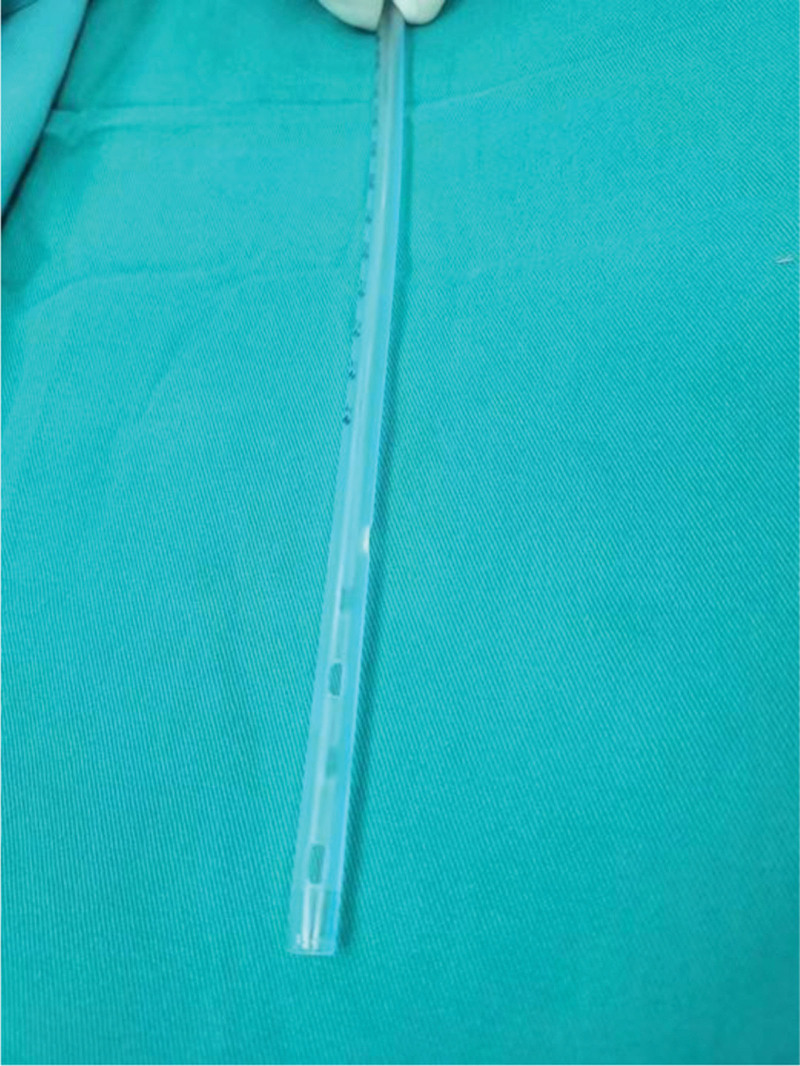
Side hole of the thoracic drain tube.

This case reports a case of hemorrhagic shock due to bleeding from a tube removal after lobectomy of the lung. After a comprehensive analysis of the condition, spontaneous bleeding due to poor coagulation was ruled out in this case, and it was finally confirmed by thoracoscopic exploratory surgery that the complication after chest drain removal was due to multiple bleeding from the pleural adhesion zone, which is extremely rare. In this case, we discussed that the most important cause of the patient’s post extubation bleeding was due to pleural adhesions, which are local adhesions between the visceral pleura and the mural pleura that occur after pleural inflammation (Fig. 5),^[10]^ which are often troublesome in lung surgery, and pleural adhesions, which can also increase the blood flow beneath the mural pleura,^[7]^ increasing the risk of bleeding. Separation of pleural adhesions during surgery is often necessary, and after separation, because of the negative chest pressure and the suction caused by the lung reopening into the side hole of the chest drain (as shown in Fig. 6), at the moment of removing the chest drain, tearing through the adhesion band of the chest wall leads to bleeding from the adhesion arteries, which, because of its large volume and duration, and the difficulty of stopping the bleeding by normal hemostasis, may result in the symptom of hemorrhagic shock.^[11,12]^

The limitations of our study include a relatively small sample size. The treatment strategy in this review was based on a sole single-center experience, with a single included case and a postoperative follow-up period of limited duration. Nevertheless, given the exceedingly low prevalence of this adverse event, it is our hope that this paper will offer some constructive insights for clinical practitioners.

**Figure 7.**
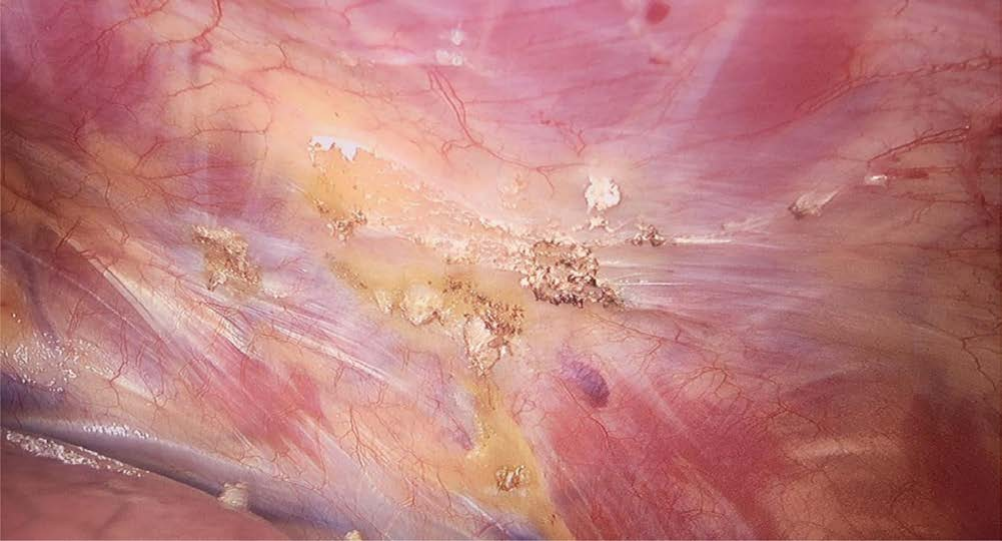
Pleural adhesions should be kept as close as possible to the chest wall.

Therefore, we propose the following recommendations for the relatively rare case of bleeding caused by removal of a chest drain after lung surgery: first, even when removing a drain that was safely inserted intraoperatively, the thoracic surgeon must be aware of possible vascular hemorrhage, and a chest drain often causes chest pain due to compression of the intercostal nerves or friction on the pleura, etc,^[13]^ but there are usually no symptoms of chest pain after the removal of a chest drain, and this The patient had heavier chest pain symptoms after the tube was removed, which was different from regular chest pain, and was most likely due to chest pain caused by vascular hemorrhage, and we should pay attention to the patient’s unusual symptoms after the operation, and keep an eye on the patient’s condition to prevent the patient’s condition from worsening. Secondly, the patient’s bleeding in this case was caused by the pleural adhesion bands implicating the blood vessels when the chest drain was removed, so we should pay attention to patiently and carefully dissecting the pleural adhesion during the operation as shown in Figure 7, and the pleural adhesion bands should be cut off by electrocautery or ultrasonic knife as close as possible to the side of the chest wall, so that there is no residual end of the adhesion bands in the thoracic cavity and the drain should be placed with a suitable shape and diameter, and the position should be far away from the adhesion bands when placing. The position should be far away from the adhesion band. Finally, when removing the chest drain, it should be rotated 360° to 720° so that it can be detached from the adhesion band or other tissues in the thoracic cavity to prevent bleeding and other adverse events.

## Author contributions

**Formal analysis:** Bin Zhang.

**Funding acquisition:** Ziteng Zhang.

**Investigation:** Baoyu He.

**Methodology:** Bin Zhang.

**Project administration:** Qichen Liang.

**Resources:** Qichen Liang.

**Software:** Baoyu He.

**Writing – original draft:** Qichen Liang.

**Writing – review & editing:** Baoyu He, Bin Zhang, Ziteng Zhang.
